# Thrombus in Transit Following Hemodynamic Stabilization in Acute Pulmonary Embolism: A Therapeutic Dilemma

**DOI:** 10.7759/cureus.114271

**Published:** 2026-08-10

**Authors:** Luis Pablo Herrera Villalba, Lilian Eréndira Pacheco Magaña

**Affiliations:** 1 Internal Medicine Department, Hospital General Regional No. 1 Morelia-Charo, Instituto Mexicano del Seguro Social (IMSS), Charo, MEX; 2 Hospital Epidemiological Surveillance Unit, Hospital General Regional No. 1 Morelia-Charo, Instituto Mexicano del Seguro Social (IMSS), Charo, MEX

**Keywords:** acute pulmonary embolism, mcconnell sign, right heart thrombus, right ventricular dysfunction, systemic thrombolysis, tapse/pasp ratio, thrombus in transit

## Abstract

Risk stratification and therapeutic decision-making in acute pulmonary embolism (PE) remain challenging, particularly in patients with apparent hemodynamic stability after an initially unstable presentation. We present the case of a 61-year-old woman with a history of type 2 diabetes mellitus, hypertension, and obesity who presented with sudden-onset dyspnea and chest pain accompanied by initial hemodynamic instability and elevated lactate levels. Computed tomography pulmonary angiography confirmed bilateral PE. Although the patient subsequently achieved hemodynamic stabilization, transthoracic echocardiography demonstrated severe right ventricular dysfunction, McConnell's sign, a right heart thrombus in transit, and right ventricle-pulmonary artery uncoupling with a tricuspid annular plane systolic excursion (TAPSE)/pulmonary artery systolic pressure (PASP) ratio of 0.19. Given these findings, systemic thrombolysis was performed, resulting in clinical and echocardiographic improvement without major bleeding complications. This case highlights the limitations of hemodynamic stability as the sole criterion for risk assessment in acute PE. The presence of a right heart thrombus in transit and right ventricle-pulmonary artery uncoupling may identify patients at increased risk of deterioration despite the absence of persistent shock. Integrating clinical and echocardiographic findings may improve the recognition of concealed high-risk features and support individualized therapeutic decision-making in acute PE.

## Introduction

Pulmonary embolism (PE) is an important cause of cardiovascular morbidity and mortality, with a broad clinical spectrum ranging from mild presentations to obstructive shock and an estimated annual incidence of approximately one case per 1,000 persons [1]. Despite advances in diagnosis and treatment, PE continues to carry a substantial burden of morbidity and mortality, as a significant proportion of patients die within the first 90 days after the event. Its consequences are not limited to the acute phase, since nearly half of patients may develop functional limitation or exercise intolerance at one year, a condition described as post-PE syndrome [1]. In Mexico, the available information comes mainly from hospital-based series, in which a high proportion of patients with right ventricular dysfunction and a mortality rate close to 14% have been reported [2]. This likely reflects both the severity of cases treated in referral centers and the limitations in timely diagnosis.

In this context, therapeutic decision-making can be complex, particularly in dynamic clinical scenarios such as initial hemodynamic instability followed by subsequent stabilization, delayed diagnosis, or previously suboptimal treatment. The 2019 European Society of Cardiology classification groups patients with intermediate-high-risk PE into a broad category that includes a heterogeneous clinical spectrum, which may hinder the identification of those at higher risk of hemodynamic deterioration who could potentially benefit from advanced therapies such as systemic thrombolysis [3]. In contrast, recent American Heart Association (AHA) and American College of Cardiology (ACC) recommendations propose a clinical category system (A-E) that integrates clinical evolution with structural findings and biomarkers, allowing a more dynamic approach to risk stratification. However, the evidence supporting its impact on therapeutic decision-making remains limited [4].

Within this scenario, PE with a right heart thrombus in transit represents an uncommon but highly unstable variant [4,5]. The coexistence of right heart thrombus and PE is associated with a worse prognosis than isolated right heart thrombus. Patients with concomitant PE have substantially higher 90-day mortality than those without PE, supporting the need for close monitoring and consideration of more aggressive therapeutic strategies in selected patients, particularly when right ventricular dysfunction or previous clinical instability is present [5].

In a systematic review and meta-analysis, the pooled prevalence of this finding was estimated at 8.1% among patients with PE [6]. However, other sources report considerable variability, ranging from 2% to 4% in recent guidelines [4] and up to 12.6% in tertiary-care hospital series in Mexico [2]. This variability probably reflects differences in patient selection and the severity of the included cases. In the same review, obstructive shock was documented in 48.9% of patients, while syncope or loss of consciousness was the initial manifestation in approximately 28.4% [6].

The optimal treatment of right heart thrombus in transit associated with PE remains uncertain, with strategies ranging from anticoagulation alone to advanced therapies such as systemic thrombolysis, surgical embolectomy, or endovascular interventions, although the absence of randomized clinical trials limits the strength of these conclusions [7]. Therapeutic management in published reviews is heterogeneous, including systemic thrombolysis in 43.8% of cases, surgical thrombectomy in 32.7%, and percutaneous thrombectomy in 6.5%, while up to 15.8% of patients received anticoagulation alone [6]. These approaches were associated with an overall mortality of 20.4%, underscoring the aggressive nature of this entity.

It is important to highlight that most of the available evidence has been generated under previous risk stratification schemes; therefore, its applicability to the new ACC/AHA proposed classifications remains uncertain. We present the case of a patient with PE who was initially unstable and subsequently stabilized, classified under traditional schemes as intermediate-high risk and corresponding to category D1R in the newly proposed stratification, which identifies a higher-severity subgroup not specifically differentiated in previous classifications [4]. In this context, the presence of a thrombus in transit raised a relevant therapeutic dilemma regarding the indication for reperfusion strategies.

## Case presentation

A 61-year-old woman with a history of type 2 diabetes mellitus, systemic arterial hypertension, and obesity developed sudden-onset dyspnea and chest pain in August 2025. She was admitted to the emergency department the next day at 4:45 a.m. because of respiratory distress.

On arrival, she had hemodynamic instability, with a blood pressure of 81/60 mmHg, a heart rate of 123 beats/min, a respiratory rate of 24 breaths/min, and an oxygen saturation of 91% while receiving an FiO₂ of 45%. Laboratory findings are summarized in Table 1. Despite these findings, the initial diagnosis was congestive heart failure, and treatment with diuretics was started without therapeutic anticoagulation.

**Table 1:** Relevant laboratory findings during hospitalization and follow-up ANCA: antineutrophil cytoplasmic antibodies; HCO₃⁻: bicarbonate; NT-proBNP: N-terminal pro-B-type natriuretic peptide; pCO₂: partial pressure of carbon dioxide; pO₂: partial pressure of oxygen

Laboratory parameter	Hospital day	Result	Unit	Reference range
pH	Hospital day 1, admission	7.400	-	7.350-7.450
pCO₂		20.00	mmHg	33.00-39.00
pO₂		64.00	mmHg	74.00-78.00
HCO₃⁻		12.40	mmol/L	18.00-23.00
Base excess		-12.40	mmol/L	-3.00 to 1.50
Lactate		9.5	mmol/L	0.5-2.2
Arterial oxygen saturation		92.00	%	92.00-96.00
Hemoglobin	Hospital day 1	15.5	g/dL	12.0-16.0
Hematocrit		48.2	%	38.0-47.0
Leukocytes		23.2	×10³/µL	5.0-10.0
Neutrophils		72.6	%	40-70
Lymphocytes		17.5	%	20-40
Platelets		221	×10³/µL	150-400
Glucose		254	mg/dL	70-100
Urea		66.34	mg/dL	18-50
Blood urea nitrogen		31	mg/dL	7-18
Creatinine		1.0	mg/dL	0.7-1.5
Sodium		139	mmol/L	135-145
Potassium		3.70	mmol/L	4.10-5.60
C-reactive protein		50.7	mg/L	<10
NT-proBNP		5729	ng/L	0-125
Creatine phosphokinase		26	U/L	0-170
Creatine kinase-MB		14.0	U/L	0-24
Prothrombin time	Hospital day 1, follow-up	10.8	s	12-16
International normalized ratio		1.00	-	0.8-1.2
Activated partial thromboplastin time		27.6	s	23-35
Fibrinogen		489	mg/dL	200-400
D-dimer		5712	ng/mL	0-500
Troponin I/T		≤0.03	ng/mL	0-0.15
pH		7.520	-	7.350-7.450
pCO₂		26.00	mmHg	33.00-39.00
pO₂		66.00	mmHg	74.00-78.00
HCO₃⁻		21.20	mmol/L	18.00-23.00
Lactate		1.3	mmol/L	0.5-2.2
Prothrombin time	Hospital day 2	10.9	s	12-16
International normalized ratio		1.01	-	0.8-1.2
Activated partial thromboplastin time		27.6	s	23-35
Fibrinogen		515	mg/dL	200-400
Total bilirubin		1.10	mg/dL	0.50-1.50
Albumin		3.10	g/dL	3.50-5.00
Alanine aminotransferase		102	U/L	0-35
Aspartate aminotransferase		39	U/L	0-37
Lactate dehydrogenase		387	U/L	120-246
D-dimer		5943	ng/mL	0-500
Hemoglobin	Hospital day 2, follow-up	14.1	g/dL	12.0-16.0
Leukocytes		13.3	×10³/µL	5.0-10.0
Platelets		151	×10³/µL	150-400
Glucose		234	mg/dL	70-100
Creatinine		0.9	mg/dL	0.7-1.5
Procalcitonin		≤0.20	ng/mL	0-0.36
NT-proBNP		5126	ng/L	0-125
pH		7.500	-	7.350-7.450
pCO₂		31.00	mmHg	33.00-39.00
pO₂		44.00	mmHg	74.00-78.00
HCO₃⁻		24.20	mmol/L	18.00-23.00
Lactate		2.3	mmol/L	0.5-2.2
Creatine phosphokinase	Hospital day 3	20	U/L	0-170
Creatine kinase-MB		12.0	U/L	0-24
Troponin I/T		≤0.03	ng/mL	0-0.15
Hemoglobin	Hospital day 4	12.1	g/dL	12.0-16.0
Leukocytes		7.5	×10³/µL	5.0-10.0
Platelets		140	×10³/µL	150-400
Creatinine		0.6	mg/dL	0.7-1.5
Albumin		2.90	g/dL	3.50-5.00
Alanine aminotransferase		96	U/L	0-35
Aspartate aminotransferase		35	U/L	0-37
Troponin I/T		≤0.03	ng/mL	0-0.15
Rheumatoid factor		8.6	IU/mL	<12
Perinuclear ANCA		<2.0	UR/mL	Negative <20.0
Anticardiolipin IgG antibodies		2.64	U IgG-FL/mL	Negative <12.0
Lupus anticoagulant screen ratio		1.21	ratio	Normal ≤1.2
Lupus anticoagulant LA1		53.4	s	31-44
Lupus anticoagulant LA2		44.3	s	30-38
Hemoglobin	Hospital day 5	11.9	g/dL	12.0-16.0
Leukocytes		5.5	×10³/µL	5.0-10.0
Platelets		173	×10³/µL	150-400
Prothrombin time		11.7	s	12-16
International normalized ratio		1.08	-	0.8-1.2
Activated partial thromboplastin time		29.3	s	23-35
Fibrinogen		417	mg/dL	200-400
Creatinine		0.6	mg/dL	0.7-1.5
Beta-2 glycoprotein IgG		<2.0	UR/mL	Negative <20.0
Protein C activity		84.4	%	70-140
Protein S activity		95.6	%	63-135
pH	Hospital day 8, discharge day	7.540	-	7.350-7.450
pCO₂		26.00	mmHg	33.00-39.00
pO₂		75.00	mmHg	74.00-78.00
HCO₃⁻		22.20	mmol/L	18.00-23.00
Lactate		0.9	mmol/L	0.5-2.2
Hemoglobin		12.9	g/dL	12.0-16.0
Leukocytes		9.8	×10³/µL	5.0-10.0
Platelets		270	×10³/µL	150-400
Creatinine		0.7	mg/dL	0.7-1.5
Perinuclear ANCA		<2.0	UR/mL	Negative <20.0

**Figure 1:**
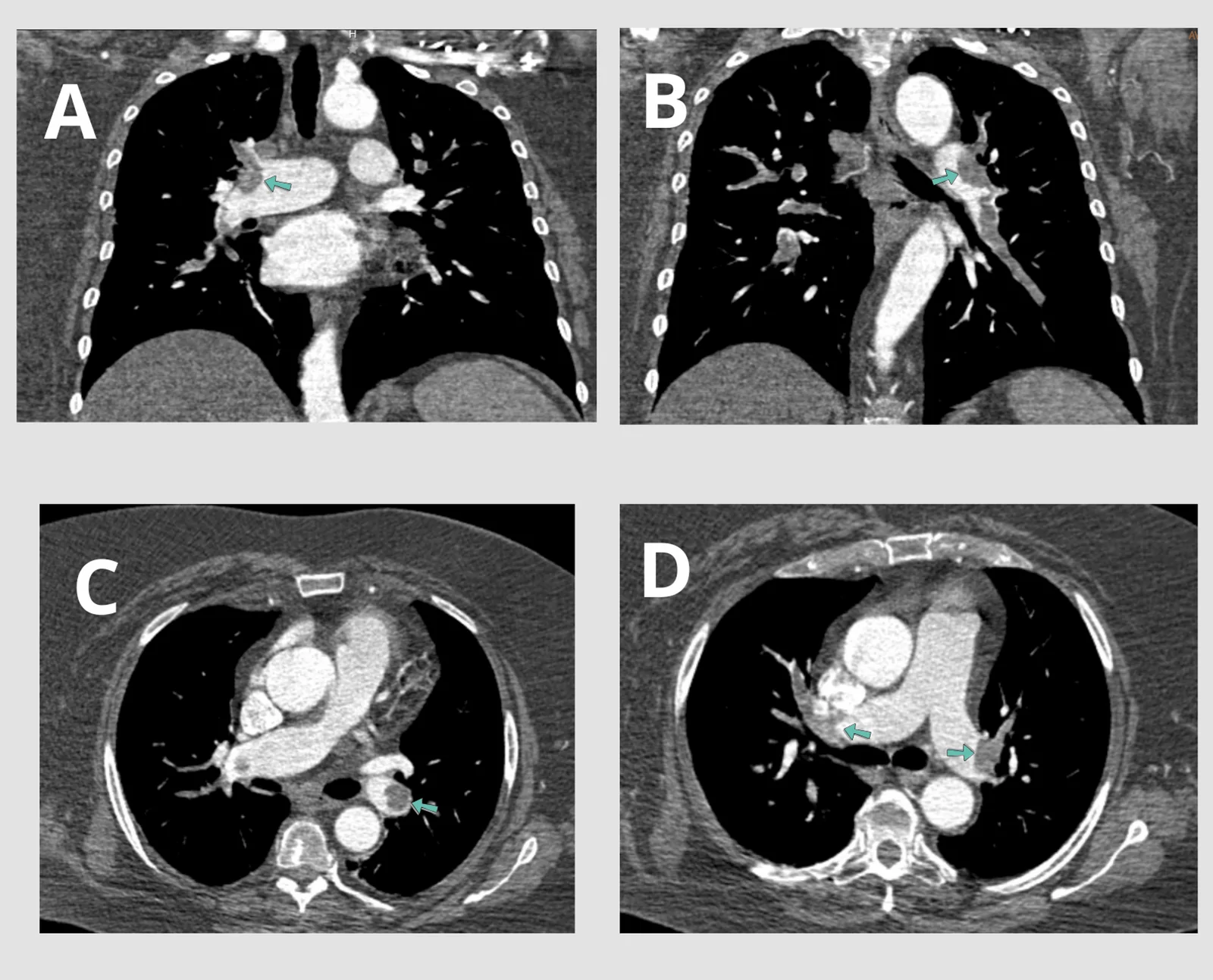
Contrast-enhanced computed tomography pulmonary angiography of the chest A and B (coronal views): Multiplanar reconstructions showing intraluminal filling defects (arrows) consistent with thrombotic material in the branches of the right and left pulmonary arteries. C and D (axial views): Transverse sections at the level of the main pulmonary artery. A marked hypodense obstructive filling defect is identified in the left main pulmonary artery (C, arrow), along with simultaneous bilateral filling defects (D, arrows), findings compatible with bilateral pulmonary embolism.

During the first 24 hours of hospitalization, treatment remained suboptimal, including enoxaparin at nontherapeutic doses. A 12-lead electrocardiogram showed complete right bundle branch block. On hospital day 2, computed tomography pulmonary angiography demonstrated bilateral PE with significant pulmonary arterial involvement (Figure 1).

On hospital day 3, after admission to the Internal Medicine department, anticoagulation was adjusted to full therapeutic dosing. Although the patient was hemodynamically stable at that time, transthoracic echocardiography demonstrated severe right ventricular dysfunction, McConnell's sign, and the presence of a right heart thrombus in transit. Right ventricle-pulmonary artery uncoupling was also documented, with a tricuspid annular plane systolic excursion (TAPSE)/pulmonary artery systolic pressure (PASP) ratio of 0.19 (Video 1).

**Video 1:** Initial transthoracic echocardiography showing severe right ventricular dilatation and a mobile thrombus in transit Two-dimensional transthoracic echocardiography demonstrating severe right ventricular dilatation, McConnell's sign, and a mobile right heart thrombus in transit measuring 0.69×0.66 cm.

Given these findings, together with the history of initial hemodynamic instability, high oxygen requirements through a simple face mask, and a Pulmonary Embolism Severity Index (PESI) score of 141 points, systemic thrombolysis with alteplase was performed at 3:00 p.m. on the same day, using a total dose of 100 mg infused over two hours. During the infusion, the patient developed mild self-limited gingival bleeding, without other hemorrhagic events.

After thrombolysis, progressive clinical improvement was observed, with decreasing oxygen requirements and sustained hemodynamic stability. Follow-up echocardiography at 24 hours showed the disappearance of the intracavitary thrombus and improvement in right ventricle-pulmonary artery coupling, with a TAPSE/PASP ratio of 0.47, although right ventricular dilation persisted (Video 2).

**Video 2:** Follow-up transthoracic echocardiography 24 hours after systemic thrombolysis Two-dimensional transthoracic echocardiography performed 24 hours after systemic thrombolysis, showing the complete resolution of the right heart thrombus in transit and a marked reduction in right ventricular diameter.

**Figure 2:**
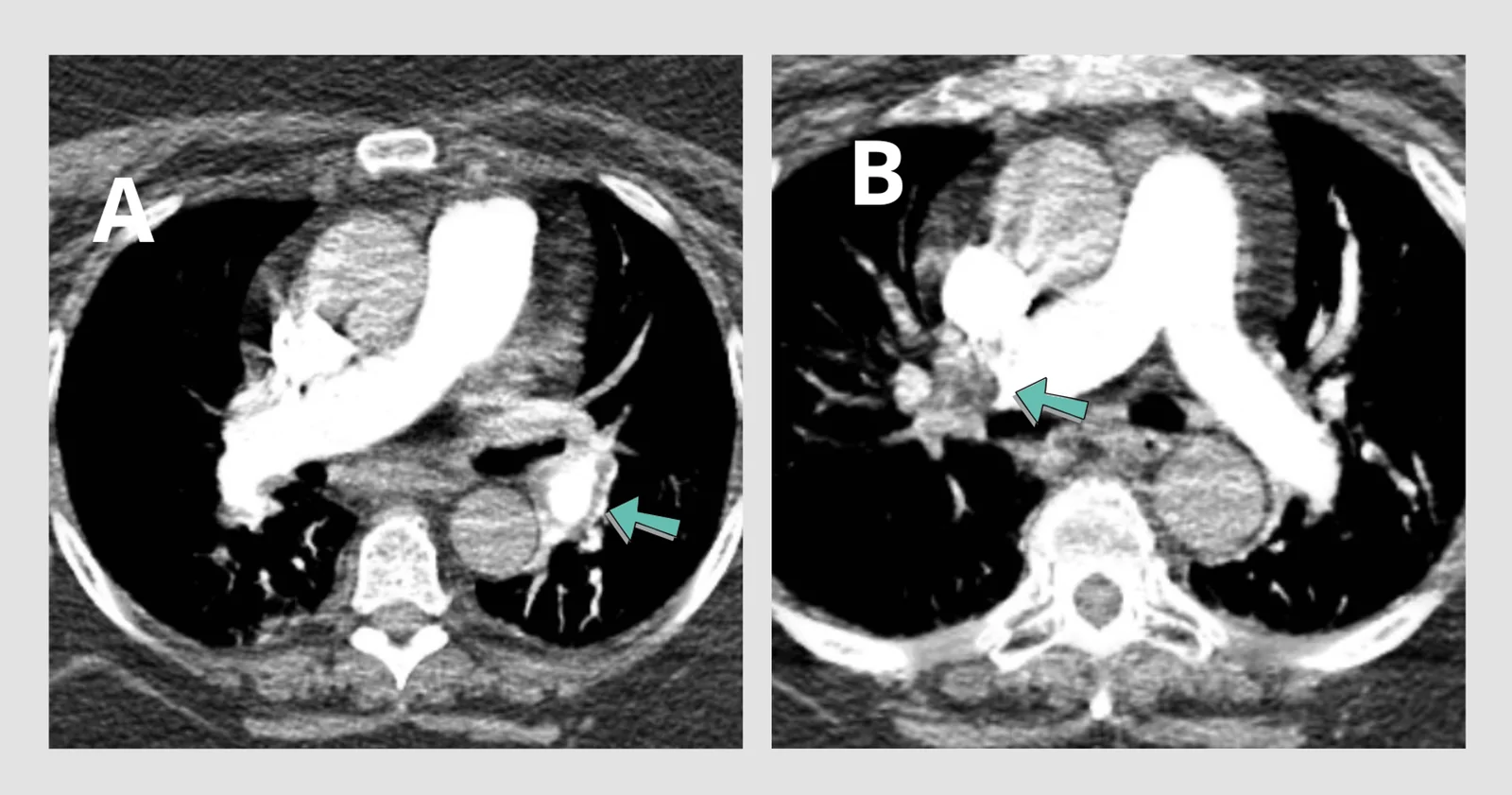
Contrast-enhanced computed tomography pulmonary angiography of the chest performed 24 hours after systemic thrombolysis A and B (axial views): Reconstructions showing a reduction in thrombotic burden within the main pulmonary arterial branches (arrows).

Follow-up computed tomography pulmonary angiography performed 24 hours after systemic thrombolysis showed a considerable reduction in thrombotic burden (Figure 2).

The patient continued to improve clinically, with progressive reduction of oxygen support, achieving low-flow nasal cannula oxygen by hospital day 6. She was discharged on hospital day 8, in good general condition, with adequate oxygenation on room air and no evidence of hemodynamic compromise. Anticoagulation with rivaroxaban was continued.

**Figure 3:**
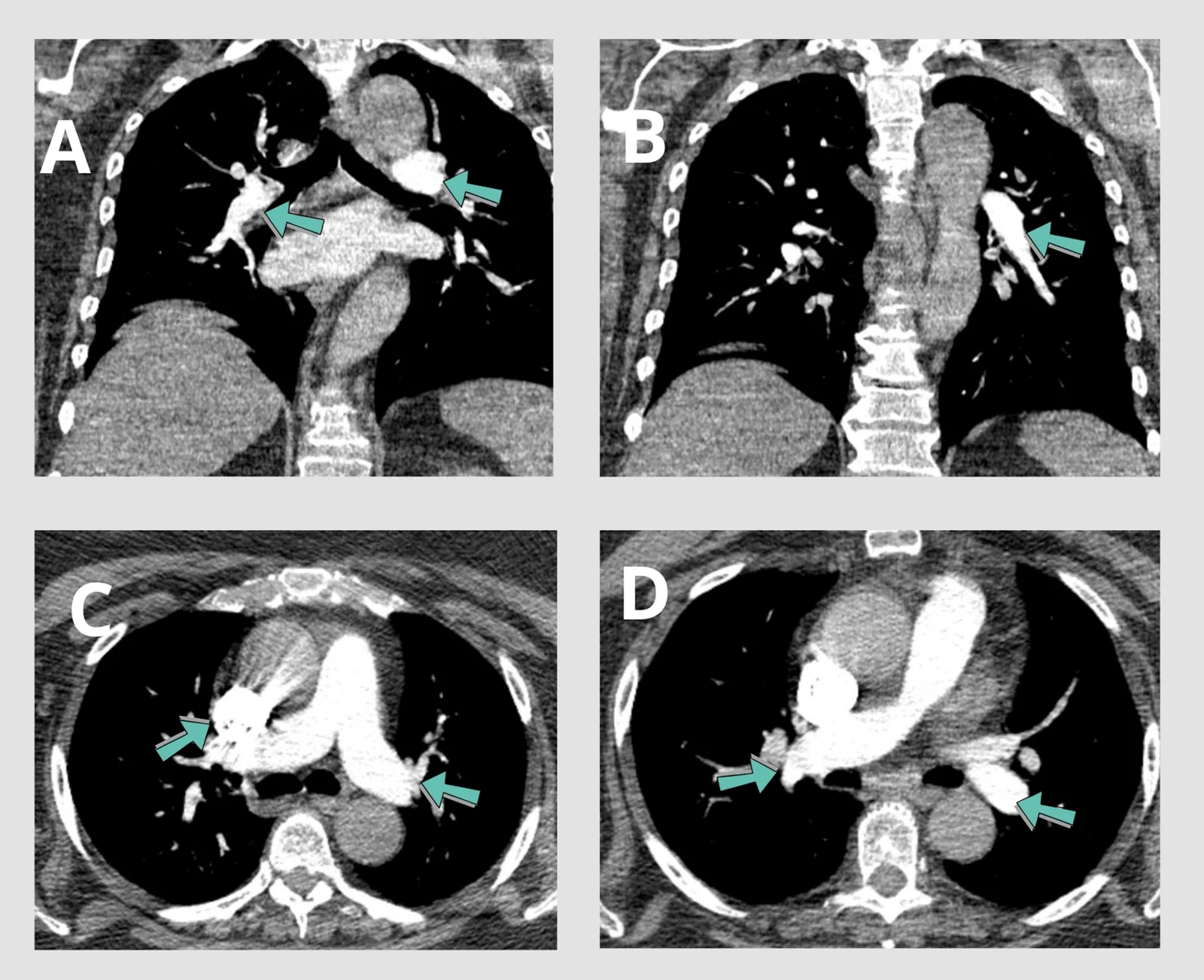
Six-month follow-up computed tomography pulmonary angiography after anticoagulation Follow-up contrast-enhanced computed tomography pulmonary angiography of the chest performed after six months of anticoagulation. A and B (coronal views): Multiplanar coronal reconstructions showing adequate opacification and patency of the right and left pulmonary arteries (arrows). C and D (axial views): Transverse sections at the level of the main pulmonary artery and its main branches. No intraluminal filling defects are identified in any projection, confirming the complete resolution of pulmonary embolism (arrows).

During follow-up, subsequent imaging studies showed no evidence of residual PE (Figure 3), and follow-up echocardiography demonstrated recovery of right ventricular function and absence of intracavitary thrombi.

## Discussion

PE is a clinical entity with a broad spectrum of presentation, ranging from subclinical forms to obstructive shock. In the present case, the patient initially presented with hypotension and markedly elevated lactate levels, consistent with obstructive shock. However, PE was not considered in the initial diagnostic evaluation, delaying therapeutic anticoagulation, a factor associated with worse clinical outcomes.

Although patients with high-risk PE often present with elevated biomarkers, their absence does not exclude severity, since risk classification is based primarily on the presence of hemodynamic instability [3]. In this case, echocardiographic evidence of right ventricular dysfunction, together with episodes of hypotension, retrospectively supports a high-risk event, corresponding to categories D1-D2 according to the AHA and ACC stratification [4]. However, under previous risk stratification schemes, the patient was classified as intermediate-high risk [3], highlighting the limitations of traditional models in capturing the full spectrum of disease severity.

Cardiac biomarkers have prognostic value in PE, but their interpretation should be integrated with clinical and echocardiographic findings. Elevation of conventional troponin T and high-sensitivity troponin T has been associated with increased short-term mortality, whereas elevation of troponin I has been associated with higher PE-related mortality. These findings reinforce the concept that myocardial injury secondary to right ventricular overload is a relevant component of clinical progression [8]. NT-proBNP has also shown prognostic utility in PE, as elevated values are associated with a higher risk of short-term clinical complications and long-term mortality. In this context, elevated NT-proBNP may support the presence of right ventricular strain even when troponin is negative; therefore, the absence of troponin elevation should not exclude disease severity when echocardiographic evidence of right ventricular dysfunction is present [8].

A particularly noteworthy feature of this case was the absence of troponin elevation despite severe right ventricular dysfunction and initial obstructive shock. This phenomenon may be explained both by analytical limitations, particularly with conventional point-of-care assays, which are the tests available at our center, and by the pathophysiology of myocardial injury in PE, which is predominantly mediated by an imbalance between oxygen supply and demand rather than by overt myocardial necrosis [9]. In this context, the use of high-sensitivity troponin assays could improve the detection of subclinical myocardial injury. The observed lactate elevation was probably multifactorial, reflecting both systemic hypoperfusion and hypoxemia, thereby reinforcing the presence of significant hemodynamic compromise. Taken together, these findings illustrate a discordance between biomarkers and structural involvement, underscoring the need for an integrated approach to risk stratification. 

A critical point in this case was the patient's subsequent apparent hemodynamic stabilization, which created uncertainty regarding the indication for systemic thrombolysis. In hemodynamically stable patients, current guidelines do not recommend routine thrombolysis because of the risk of bleeding [3,4]. However, this approach may be insufficient in scenarios in which high-risk markers are present but not reflected by clinical stability alone. In this context, the decision to perform thrombolysis was supported by the presence of severe right ventricular dysfunction, an intracavitary thrombus, and evidence of right ventricle-pulmonary artery uncoupling, with a TAPSE/PASP ratio of 0.19. This parameter has been independently associated with clinical deterioration at 48 hours (hazard ratio: 2.06) and 30-day mortality (hazard ratio: 2.28) [10]. Additionally, a TAPSE/PASP ratio of <0.4 predicts hemodynamic deterioration and mortality in acute PE, emphasizing that transient hemodynamic stability alone may underestimate disease severity [11].

An important aspect is that current PE guidelines do not formally incorporate right heart thrombus in transit into conventional risk stratification schemes. Therefore, some authors propose considering it a marker of more severe disease and using it as a factor to lower the threshold for advanced therapies, especially when right ventricular dysfunction and high thrombus mobility coexist, because of the risk of further embolization and abrupt hemodynamic deterioration [7]. The presence of thrombus in transit is associated with high mortality regardless of the therapeutic strategy, with reported mortality rates of 28.6% for anticoagulation, 23.8% for surgical treatment, and 11.3% in patients treated with thrombolysis [11]. These findings reinforce the need for individualized decision-making.

Catheter-directed therapies have recently emerged as an alternative in patients with intermediate-high-risk and high-risk PE, particularly when there is a relative contraindication to systemic thrombolysis or when the goal is to reduce thrombotic burden with lower systemic exposure to fibrinolytic agents. Recent meta-analyses have suggested an association between these strategies and lower mortality compared with anticoagulation alone, although much of the evidence comes from observational studies and should therefore be interpreted with caution [12]. Nevertheless, the applicability of these strategies depends on institutional availability, center experience, and the ability to activate multidisciplinary teams with access to vascular intervention, cardiothoracic surgery, or specialized PE response teams. In the present case, because catheter-directed therapies and surgical embolectomy were not immediately available, systemic thrombolysis represented the most accessible and timely reperfusion strategy in the setting of severe right ventricular dysfunction, thrombus in transit, and a history of initial hemodynamic instability [7].

After thrombolysis, the patient showed clinical and echocardiographic improvement, with resolution of the intracavitary thrombus and progressive recovery of right ventricular function. Although she developed mild gingival bleeding, this did not require intervention, suggesting that, in selected scenarios, the benefit of thrombolysis may outweigh its risks. Finally, complete resolution on follow-up imaging supports the effectiveness of the therapeutic approach used in this case and reinforces the hypothesis that a strategy guided by structural and functional parameters may be decisive in selected subgroups.

## Conclusions

Risk stratification in PE should not be based exclusively on hemodynamic stability. This case demonstrates that the integration of clinical, echocardiographic, and functional findings can help identify patients with "concealed" high-risk features who may benefit from reperfusion strategies.

In particular, right ventricle-pulmonary artery uncoupling and the presence of a thrombus in transit emerge as variables with potential prognostic and therapeutic value beyond traditional algorithms. These findings highlight the limitations of conventional classifications and support the usefulness of more recent models for improved risk characterization. The favorable clinical course observed in this case suggests that, in selected patients, an individualized strategy based on advanced echocardiographic parameters and clinical findings may contribute to more precise therapeutic decision-making.
